# Supplementary material for: Ameliorating impact of coenzyme Q10 on the profile of adipokines, cardiomyopathy, and hematological markers correlated with the glucotoxicity sequelae in diabetic rats
Source: PLoS One. 2024 Jan 16;19(1):e0296775. doi: 10.1371/journal.pone.0296775 (PMC10790996; doi:10.1371/journal.pone.0296775)
Supplement: Attachment — Submitted filename: Response to Reviewers.docx [file pone.0296775.s002.docx]

**Rebuttal letter**

**Response to reviewers**

We express our gratitude to the editor and reviewers for their evaluation of our manuscript. Based on the feedback provided by the reviewers, we have implemented revisions to the manuscript, resulting in what we believe is a considerable improvement.

We will provide a detailed response to each comment or question, addressing them point by point. Please follow our responses accordingly:

**Reviewer 2**

We wish to express our sincere gratitude for the valuable feedback you have shared. Your insights are immensely appreciated and will undoubtedly contribute to the improvement of our manuscript.

**1-In the abstract's background section, it would be helpful to provide a brief explanation of glucotoxicity and its relevance to diabetes mellitus. This will provide more context for readers who may not be familiar with the term.**

**Answer:** We concur with the reviewer's comment and have made changes to the content in the background section of the abstract to succinctly elucidate glucotoxicity and its relevance to diabetes mellitus. Nevertheless, to comply with the 300-word limit, we made a minor adjustment.

**2. In the abstract's conclusion section, it would be beneficial to summarize the main findings of the study concisely. Additionally, discussing the potential implications of the results and highlighting any limitations or future directions for research would be useful.**

**Answer:** We discussed the beneficial effects of the antioxidant CoQ10 that we investigated, particularly its role in improving diabetic complications. Due to the 300-word limit, we were unable to provide further explanation within our abstract. We addressed the potential implications of our study results in the final paragraph of the introduction section and in the conclusion of our article.

**3. Ensure that the references cited in the manuscript follow a consistent format. Maintain uniformity in the citation style throughout the text.**

**Answer:** Thank you for your valuable feedback regarding the consistency of citation formatting in our manuscript. We have taken your comments seriously and have made concerted efforts to ensure uniformity in the referencing style throughout the document.

To address this concern, I meticulously reviewed all references cited in the manuscript and utilized the reference management tool, EndNote, to enforce adherence to the PLOS ONE style (Vancouver). This involved a comprehensive check of each citation within the text, cross-referencing them with the bibliography or reference list, and ensuring that they align with the specified guidelines of the Vancouver style.

4. **In the methodology, please include specific details about the conditions of acclimatization and measures taken to ensure the animals' well-being during this period. This should encompass** **information such as the number of animals in each cage, environmental enrichment, and health and behavior monitoring.**

**Answer:** We appreciate your guidance on the revisions; we made changes accordingly. We specified the number of animals in each cage and also included details about specific details about the conditions of acclimatization, environmental enrichment, as well as health and behavior monitoring in Lines 166-184.

**5. Please provide more information regarding the ELISA procedure. Include details such as dilutions, incubation times, washing steps, and other relevant protocol-specific information for each ELISA kit used in the study.**

**Answer:** Thank you for your query regarding the ELISA procedure. We appreciate your interest in the details and protocol specifics.

In our manuscript, we have provided a concise overview of the ELISA procedure in lines 251-268. Additionally, we have attached the PDF file containing the Rat LP-PLA2 ELISA Kit instruction to this email for your reference. If you believe that including specific details from the kit catalogues in the study manuscript would enhance its comprehensiveness, we are more than willing to incorporate this information in our next revision. Your input is highly valued, and we aim to address all essential details to strengthen the manuscript.

Furthermore, an extensive search was conducted within the PLOS ONE journal to gather relevant information. Specifically, we tailored our procedure to align with the methodology outlined in previously published articles; the DOI of one such article is provided below:

https://doi.org/10.1371/journal.pone.0202797

**6. The manuscript would benefit from proofreading to correct minor grammatical and typographical errors, improving clarity and readability.**

**Answer:** Thank you for your constructive feedback. We acknowledge the need for proofreading to rectify any minor grammatical and typographical errors in the manuscript. Ensuring clarity and readability is pivotal, and we are committed to enhancing these aspects.

In our forthcoming revision, we will diligently conduct a thorough proofreading process to address these issues and refine the overall quality of the manuscript.

**Reviewer 3**

**The paper is well-designed and prepared. All comments have been addressed, and there are no further comments. Accept in the present form.**

Thank you for your positive feedback and acceptance recommendation. We appreciate your time and effort in reviewing our article. Your insights were invaluable.

Best regards,
